# Overlapping expression patterns and functions of three paralogous P5B ATPases in *Caenorhabditis elegans*

**DOI:** 10.1371/journal.pone.0194451

**Published:** 2018-03-16

**Authors:** Jeffrey Zielich, Elena Tzima, Eva Ayla Schröder, Faten Jemel, Barbara Conradt, Eric J. Lambie

**Affiliations:** 1 Department of Cell and Developmental Biology, Ludwig Maximilian University, Planegg-Martinsried, Munich, Germany; 2 Center for Integrated Protein Science Munich, Ludwig Maximilian University, Munich, Germany; East Carolina University, UNITED STATES

## Abstract

P5B ATPases are present in the genomes of diverse unicellular and multicellular eukaryotes, indicating that they have an ancient origin, and that they are important for cellular fitness. Inactivation of ATP13A2, one of the four human P5B ATPases, leads to early-onset Parkinson’s disease (Kufor-Rakeb Syndrome). The presence of an invariant PPALP motif within the putative substrate interaction pocket of transmembrane segment M4 suggests that all P5B ATPases might have similar transport specificity; however, the identity of the transport substrate(s) remains unknown. Nematodes of the genus Caenorhabditis possess three paralogous P5B ATPase genes, *catp-5*, *catp-6* and *catp-7*, which probably originated from a single ancestral gene around the time of origin of the Caenorhabditid clade. By using CRISPR/Cas9, we have systematically investigated the expression patterns, subcellular localization and biological functions of each of the P5B ATPases of *C*. *elegans*. We find that each gene has a unique expression pattern, and that some tissues express more than one P5B. In some tissues where their expression patterns overlap, different P5Bs are targeted to different subcellular compartments (e.g., early endosomes vs. plasma membrane), whereas in other tissues they localize to the same compartment (plasma membrane). We observed lysosomal co-localization between CATP-6::GFP and LMP-1::RFP in transgenic animals; however, this was an artifact of the tagged LMP-1 protein, since anti-LMP-1 antibody staining of native protein revealed that LMP-1 and CATP-6::GFP occupy different compartments. The nematode P5Bs are at least partially redundant, since we observed synthetic sterility in *catp-5(0); catp-6(0)* and *catp-6(0) catp-7(0)* double mutants. The double mutants exhibit defects in distal tip cell migration that resemble those of *ina-1* (alpha integrin ortholog) and *vab-3* (Pax6 ortholog) mutants, suggesting that the nematode P5Bs are required for *ina-1*and/or *vab-3* function. This is potentially a conserved regulatory interaction, since mammalian ATP13A2, alpha integrin and Pax6 are all required for proper dopaminergic neuron function.

## Introduction

**Fig 1:**
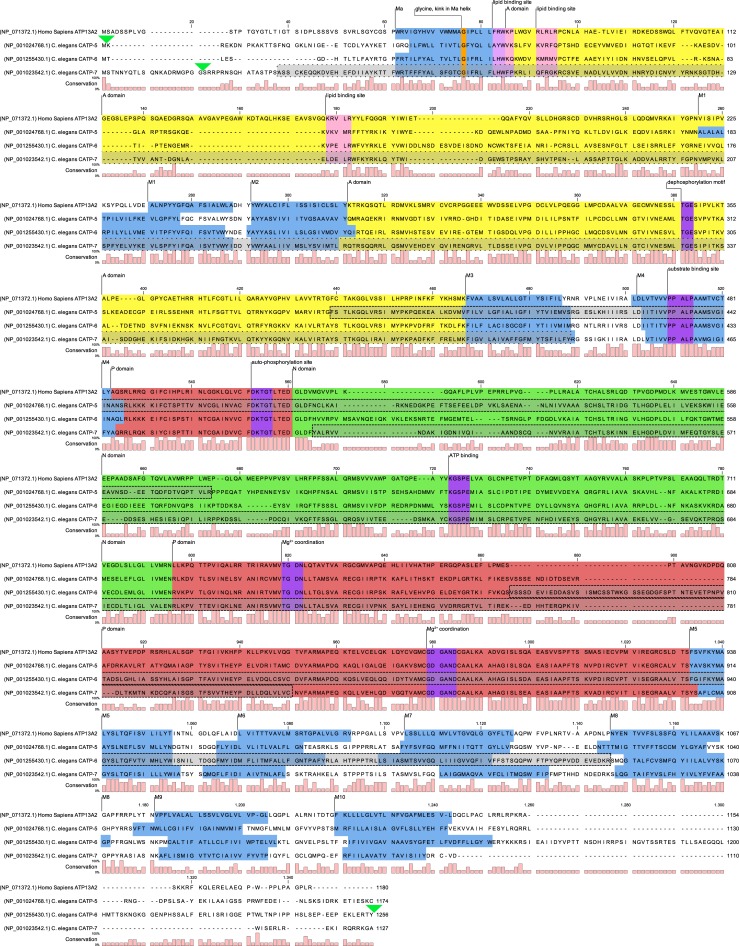
*C*. *elegans* P5B sequence alignment. Protein sequence alignment of *C*. *elegans* P5B ATPases in comparison with Human ATP13A2. Blue: Putative membrane associated domain Ma [9] and putative transmembrane domains M1–M10 (TMHMM v1.6). Yellow: A (actuator) domain. Red: P (phosphorylation) domain. Green: N (nucleotide binding) domain [8]. Orange: Putative kink in Ma through conserved glycine. Pink: Putative lipid binding site. Purple: P-type ATPase motifs [9]. Green triangle: Fluorescent protein insertion site via CRISPR/Cas9. Dashed grey boxes: *catp-5(tm4481)*, *catp-6(ok3473)* and *catp-7(tm4438)* respectively. Dotted grey box: *catp-7(dx189)* CRISPR/Cas9 mediated deletion.

**Fig 2:**
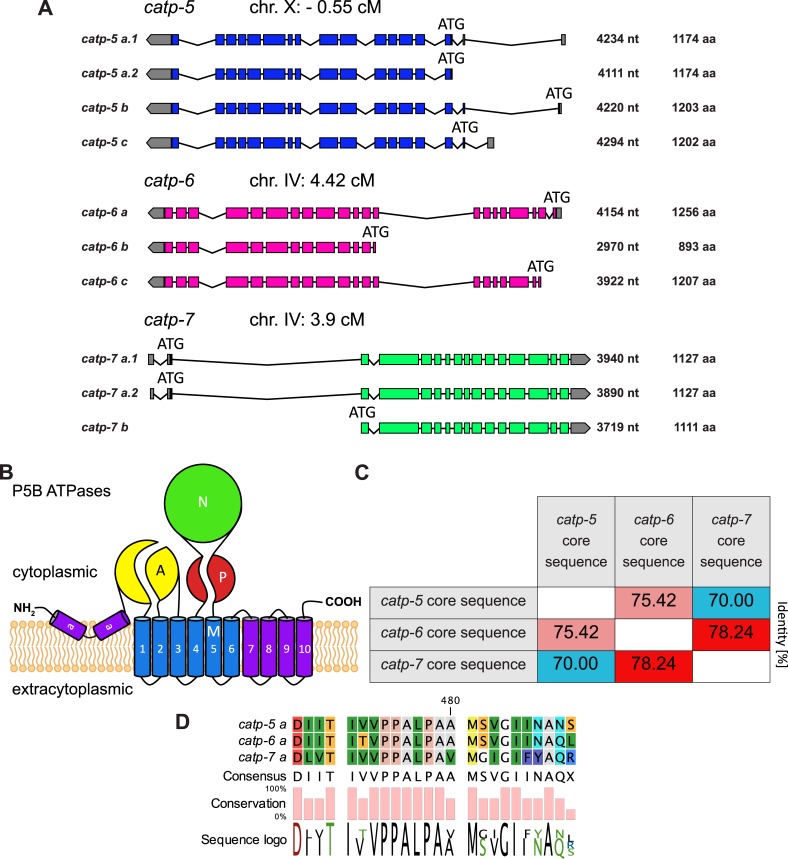
Paralogous P5B ATPases of *C*. *elegans*. (A) Genomic locations including transcripts, transcript length (in nucleotides, nt) and protein length (in amino acids, aa) of *catp-5*, *catp-6* and *catp-7* (WormBase WS262). (B) Schematic of the general structure of P5B ATPases. The A (actuator) domain, the P (phosphorylation) domain, the N (nucleotide binding) domain and the M (transmembrane M1–M10) domain plus the additional membrane associated (Ma) domain are indicated [9]. (C) Similarity matrix of CATP-5, CATP-6 and CATP-7 core sequences. The core sequences consists of 239 aa, according to [7]. (D) Comparison of the amino acid sequences of M4.

P-type ATPases are an ancient family of transmembrane proteins that use the energy derived from hydrolysis of ATP to actively transport substrates across membranes [1]. These transporters have four types of structural domains (Fig 1, Fig 2B): the actuator domain (A), the nucleotide binding domain (N), the phosphorylation domain (P) and the transmembrane domain (M) [2]. The signature characteristic of P-type ATPases is the highly conserved cytoplasmic DKTGT motif (P-domain, Fig 1) [1], which is autophosphorylated on aspartate during the catalytic cycle [3]. The P-type ATPases can be grouped into 5 subfamilies, P1-P5 [4]. The cellular functions and substrate specificities have been defined for one or more representatives of each of the P1-P4 subfamilies; however, specific substrates have not been definitively determined for either of the P5 subgroups, P5A and P5B [2,5–7]. In this study, we focus on the P5B P-type ATPases, which have a putative substrate interaction motif of PPALP within transmembrane segment M4 [7,8].

The *C*. *elegans* genome encodes three P5B ATPases: CATP-5, CATP-6 and CATP-7. These proteins have a high degree of similarity, particularly in the M4 transmembrane domain (Fig 1, Fig 2D), which is thought to be critical for coordinating substrate in the binding pocket formed by M4, M5, M6, M8 and M9 [10] (Fig 1, Fig 2B). This suggests that CATP-5, CATP-6 and CATP-7 could have the same substrate specificity and therefore fulfill the same biochemical functions, but in different tissues and/or subcellular compartments.

CATP-5::GFP has been shown to localize to the apical/surface of the intestinal cells and is required for the efficient uptake of polyamines from the gut lumen [11]. We previously showed that CATP-6 localizes to vesicular structures in multiple cell types, and that it acts to promote the function of the SLC16A transporter, GEM-1 [12]. No characterization of *catp-7* has yet been reported in the literature. In this study, we use CRISPR/Cas9 to characterize all three *C*. *elegans* P5B P-type ATPases with regard to spatiotemporal expression pattern, subcellular localization and biological function in living animals.

## Material and methods

### Strains and genetics

All strains were maintained at 23.5°C on nematode growth medium (NGM) plates with *E*. *coli* strain AMA1004 [13] as food source. Bristol N2 was used as the wild-type (wt) strain [14]. Some of the mutations and genome modifications were obtained from the *Caenorhabditis* Genetics Center at the University of Minnesota (CGC, Minneapolis, MN, USA), the National Bioresource Project (University of Tokyo, Japan), and the laboratory of Dr. Barth Grant (Rutgers University, NJ, USA). The following alleles were used in this study: *xnIs459[yfp*::*ral-1a + unc-119(+)]* III [15], *catp-6(ok3473)* IV, *catp-7(tm4438)* IV [16], *catp-7(dx189[delta 1492 bp Ma-M3 + gfp + loxP]* IV, *catp-6(dx183[catp-6*::*gfp*::*3xFlag + loxP])* IV, *catp-6(dx179[catp-6*::*degron*::*mKate2*::*3xFlag + loxP])* IV, *catp-7(dx185[gfp*::*catp-7 + loxP])* IV, *catp-7(dx191[mcherry*::*catp-7 + loxP])* IV, *catp-7(dx193[gfp*::*catp-7 + loxP])* IV, *ltIs44(P*_*pie-1*_*mCherry*::*ph*^*PLCδ*^*)* V [17], *catp-5(dx187[gfp*::*catp-5 + loxP])* X, *catp-5(tm4481)* X [16], *bgIs312[pes-6*::*gfp]* [18], *xnIs484 [mCherry*::*sec-10 + unc-119(+)]* [15], *pwIS1039[p*_*snx-1*_*citrine*::*hgrs-1*_*unc-54*_ *UTR-cb-unc-119]*, *pwIs1175[p*_*snx-1*_*tgn-38*::*gfp*_*unc-54*_ *UTR-cb-unc-119]; unc-119(ed3)*, *pwIs1176[p*_*snx-1*_*ss-gfp-cd4-hcimpr*_*unc-54*_ *UTR-cb-unc-119]; unc-119(ed3)* [19].

### Protein sequence alignment

Alignments were performed by using CLC Main Workbench 8.0 (Qiagen Bioinformatics). Parameters were kept as default (gap open cost: 10, gap extension cost: 1, end gap cost: as any other). Transmembrane domains were predicted by using TMHMM 1.6 CLC Main Workbench Plugin. Domains and motifs were identified according to the literature [8,9].

### Core sequence alignment

Core sequences were identified according to [7]. Alignments were performed by using CLC Main Workbench 7.7.2 (Qiagen Bioinformatics). Parameters were kept as default (gap open cost: 10, gap extension cost: 1, end gap cost: as any other).

### Phylogenetic analysis

Sequences were obtained by using pblastp suite https://blast.ncbi.nlm.nih.gov/Blast.cgi or directly from http://flybase.org and http://www.wormbase.org. Alignment was performed by using the alignment tool of CLC Main Workbench 7.9.1 (Qiagen Bioinformatics). Parameters were kept as default (gap open cost: 10, gap extension cost: 1, end gap cost: as any other). We tested for the appropriate model by using MEGA6.06 [20]. A Maximum Likelihood tree was constructed via PhyML3.2 [21] by using the model LG+G ([22], gamma shape parameter: 0.628) and Bootstrap branch support (100 replicates). The final tree was modified by using CLC Main Workbench 7.9.1 (Qiagen Bioinformatics) and Affinity Designer 1.6.0 (Serif).

### Molecular biology

Standard methods for DNA amplification, analysis and manipulation were used. PCR products were amplified by using Phusion® High-Fidelity DNA Polymerase (New England Biolabs), according to the manufacturer's protocol. DNA sequences were obtained by Sanger sequencing.

### Plasmid construction

#### *P*_*catp-5*_*catp-5*::*gfp*_*let-858*_ and *P*_*pgp-12*_*catp-5*::*gfp*_*let-858*_

We amplified the coding sequence of *catp-5a* from the fosmid WRM0614C_D10 together with 4100 bp 5’ of exon 1 as a putative promoter. Sequence ends: 5’ ttgtctgtgacaacaacagg and 3’ agactatagagagcaaatgt (plus homology to *gfp* and backbone). We fused the coding sequence of *gfp* and the sequence of *let-858 3’UTR* in frame to the 3’ end of *catp-5a* by using Gibson cloning (backbone: pGEM-7Zf(+)) [23]. We exchanged *P*_*catp-5*_ with the putative *pgp-12* promoter [24] (amplified from wt genomic DNA, 3591 bp) via Gibson cloning. Sequence ends: 5’ ctgaagcttagcctcttcac and 3’ cttctgaaataggttaaacg (plus homology to *catp-5a* and backbone)

#### P_catp-6_catp-6::mKate2_let-858_

We amplified the coding sequence of *catp-6a* from the fosmid WRM067B_F08 together with 1135 bp 5’ of exon1 as a putative promoter. Sequence ends: 5’ gccacaataaaataataata and 3’ aaaagcttgaacgcacttac (plus homology to *mKate2* and backbone). We fused the coding sequence of *mKate2* and the sequence of *let-858 3’UTR* in frame to the 3’ end of *catp-6a* by using Gibson cloning (backbone: pGEM-7Zf(+)). We used the aa sequence GASGASGAS as a flexible linker between CATP-6 and mKate2.

#### P_catp-7_catp-7::gfp_let-858_

We amplified the coding sequence of *catp-7a* from the fosmid WRM0640A_C09 together with 2377 bp 5’ of exon1 as a putative promoter. Sequence ends: 5’ ttactttacggggtgaccct and 3’ gtcagaggcggaaaggtgct (plus homology to g*fp* and backbone). We fused the coding sequence of *gfp* and the sequence of *let-858 3’UTR* in frame to the 3’ end of *catp-7a* by using Gibson cloning (backbone: pGEM-7Zf(+)). We used the aa sequence GPGGP as a flexible linker between CATP-7 and GFP.

#### *P*_*sth-1*_*lmp-1*::*rfp*_*unc-54*_, *P*_*sth-1*_*rfp*::*rab-5*_*unc-54*_ and *P*_*sth-1*_*rfp*::*rab-11*_*unc-54*_

The coding sequences of *lmp-1* (Sequence ends: 5’ atggtgtctaagggcgaaga 3’ aactggggcacaaacttaat), *rab-5* (5’ atggccgcccgaaacgcagg 3’ taaataagaattccaactga) and *rab-11* (5’ atgggctctcgtgacgatga 3’ agcagtgttgcatcccataa) were amplified from wt genomic DNA (added homology to *Tagrfp* [25], *unc-54 3’ UTR* or *P*_*sth-1*_). We fused the putative *sth-1* promoter (*P*_*sth-1*_ 1230 bp: 5’ gaagctgaatgcgatgtctt 3’ tctttttgtgctagagcaac) [26] plus the coding sequence of *rfp* in frame to the 5’ end (or *rfp* to the 3’ end of *lmp-1*) and the sequence of *unc-54 3’UTR* to the 3’ end of *rab-*5 and *rab-11* by using Gibson cloning.

#### P_rgs-1_lmp-1::rfp_unc-54_

We amplified the putative *rgs-1* promoter (2393 bp, Sequence ends: 5’ agtaatttggcgtaagtttc 3’ ctgcgacgtgttgccgccag) [27] from wt genomic DNA with primers that provide homology to *lmp-1* for *in vivo* recombination with *lmp-1*::*rfp*_*unc-54*_.

### CRISPR/Cas9 mediated genome modifications

All sgRNAs were designed by using either the online sgRNA design tool http://crispr.cos.uni-heidelberg.de/index.html [28] or http://www.e-crisp.org/E-CRISP/ [29]. sgRNA expression plasmids were cloned by using pRB1017 following the protocol of [30]. The sgRNAs were expressed under the control of a PU6 promoter. In order to obtain large genome modifications we used two different drug-selection based methods, as recommended [31,32]. We used the following injection-mix concentrations for the self-excising cassette (SEC) drug selection method [32]: pDD162 (*P*_*eft-3*_*Cas9* expression plasmid) at 50 ng/μl, sgRNA plasmid (based on pRB1017) at 50 ng/μl, SEC repair template at 10 ng/μl (based on pDD282 and pDD285), L3790 (*P*_*myo-2*_*gfp*, Fire Lab 1995 Vector Kit) at 7 ng/μl. We used the following injection-mix concentrations for the dual marker selection cassette (DMSC) method [31]: Peft-3::Cas9_SV40_NLS::tbb-2_UTR (*P*_*eft-3*_*Cas9* expression plasmid) at 50 ng/μl, sgRNA plasmid (based on pRB1017) at 100 ng/μl, DMSC repair template at 50 ng/μl (based on loxP_myo2_neoR_GFP_intron), pCFJ90 at 2.5 ng/μl (*P*_*myo-2*_*mcherry*), pCFJ104 at 5ng/μl (*P*_*myo-3*_*mcherry*). Detailed procedures and a list of all primers used for repair template construction will be provided upon request. All genome modifications were performed in a wild type N2 background, unless stated otherwise.

#### *catp-5(dx187[gfp*::*catp-5 + loxP])* X

We targeted exon 1 of *catp-5a* by using sgRNA(catp-5#2): ctctcgcttcataattttcgtgg (underlined PAM). DMSC *gfp*-fusion repair template was constructed according to [31]. The PAM was mutated silently from tgg to tcg. The coding sequence of *gfp* + *loxP* was inserted into the genome 3’ of the following sequence: acaatccatcgacaaaaatt and 5’ of the sequence: aagcgagagaaagacaatcc.

#### *catp-6(dx183[catp-6*::*gfp*::*3xFlag + loxP])* IV and *catp-6(dx179[catp-6*::*degron*::*mKate2*::*3xFlag + loxP])* IV

We targeted the last exon of *catp-6a* by using sgRNA(catp-6#14): gttccgaaaggctgtgaggagg (underlined PAM). SEC *gfp*-fusion repair template was constructed according to [32]. The PAM was mutated from agg to aaa which changes proline 1239 to a phenylalanine (CATP-6a(P1239F)); this residue is not evolutionarily conserved. The coding sequence of *gfp*::*3xFlag* + *loxP* and a sequence that encodes a short flexible linker (ggagcatcgggagcctcaggagcatcg, GASGASGAS) was inserted into the genome 3’ of the following sequence: aaaagcttgaacgcacttac and 5’ of the sequence: aatcactttgttttagattt.

The *degron*::*mKate2* fusion was obtained by following the same procedure. But in addition we introduced the coding sequence of a *degron* motif [33] into the repair template, 5’ of the coding sequence of *mKate2* and flanked by two flexible aa linkers of the same sequence (GASGASGAS).

#### *catp-7(dx185[gfp*::*catp-7 + loxP])* IV and *catp-7(dx191[mcherry*::*catp-7 + loxP])* IV

We targeted exon 2 of *catp-7a* by using the sgRNA(catp-7#12): catgaggaggcggatgggg (underlined PAM). DMSC *gfp*-fusion and *mcherry*-fusion repair template were constructed according to [31]. The PAM was mutated silently from ggg to gcg. The coding sequence of either *gfp* + *loxP* or *mcherry* + *loxP* was inserted into the genome 3’ of the following sequence: gatcgtatgggccctggcgg and 5’ of the sequence: tctcgccgtccaagaaattc.

#### *catp-6(dx179[catp-6*::*degron*::*mKate2*::*3xFlag + loxP]) catp-7(dx193[gfp*::*catp-7 + loxP])* IV

We targeted exon 2 of *catp-7a* in the genetic background of *catp-6(dx179[catp-6*::*degron*::*mKate2*::*3xFlag + loxP])* IV, by using sgRNA(catp-7#12): catgaggaggcggatgggg (underlined PAM). *gfp* + *loxP* integration was obtained as described in the previous section.

#### *catp-6(ok3473) catp-7(dx189[delta 1492 bp Ma-M3 + gfp + loxP])* IV

We targeted exon 2 of *catp-7a* in the background of *catp-6(ok3473)* by using the following sgRNAs: sgRNA(catp-7#12): catgaggaggcggatgggg and sgRNA(catp-7#2): cgttcatccttttctaccgtggg (underlined PAM). The PAM of the first sgRNA was mutated silently from ggg to gcg. DMSC *gfp*-fusion repair template was constructed according to [31] by using homology tails that omit 1492 bp between exon 2 and exon 5. After recombination with the genome, 1492 bp between exon 2 and exon 5 were excised and *gfp + loxP* was inserted into the genome 3’ of the following sequence: cgaccgcctccaccccatcc. And 5’ of the sequence: cgtgggagttcaattggaaa. The integration led to a frameshift 3’ of *gfp*. Worms were propagated as heterozygotes (*catp-6(ok3473) catp-7(dx189)*/ *nT1[qIs51]* (IV;V)).

### Immunochemistry

Worms were dissected in M9 to release gonadal tissues. Gonads were transferred to polylysine-D-coated slides and covered with coverslips [34]. The slides were frozen on dry-ice cooled steel plates. Coverslips were removed after 10 min, slides were fixed for 10 min in -20°C methanol and thereafter transferred to -20°C acetone for 10 min. Slides were incubated in AbA + 1% Triton X-100 (PBS + 0.05% Tween-20 + 0.5% BSA + 1% Triton X-100) for 20 min [35]. Afterwards, samples were blocked for 30 min in BLOCK (PBS + 2% BSA + 2% powdered milk (Sigma) + 0.05% Tween-20). Slides were then washed for 5 min in PBST (PBS + 0.1% Tween-20). Primary antibodies were diluted 1:10 (anti-LMP-1 and anti-RME-1 [35]) and 1:500 (anti-GFP, ab290, Abcam) in BLOCK and used for incubation overnight. Next, slides were washed for 10 min in PBST. Secondary antibodies were diluted 1:500 in BLOCK and used for 1h incubation (IgG Alexa 594 anti mouse and Alexa 488 anti rabbit). Slides were washed for 5 min in PBST, then transferred to PBS for an additional 5 min. Gonads were mounted with VECTASHIELD Antifade Mounting Medium with DAPI (Vector Laboratories) and sealed with nail polish. A Leica TCS SP5 II confocal microscope was used for imaging.

### Imaging

Animals were mounted on 4% agarose pads for confocal, DIC and epifluorescence microscopy. Whole worms were immobilized with 10 mM levamisole and/or 1 mM sodium azide. Animals were imaged by using either Leica TCS SP5 II confocal microscope (Leica Application Suite LAS software) or Zeiss Axioskop 2 and MetaMorph software (Molecular Devices). Image processing was performed in Fiji/imageJ 2.0.0 [36], brightness and contrast was adjusted either in Fiji/imageJ 2.0.0 or Affinity Designer 1.6.0 (Serif).

### Fluorescence Recovery after Photobleaching (FRAP)

A Leica TCS SP5 II confocal microscope was used for imaging and photobleaching. We performed photobleaching for 1 min (laser intensity change from 6% to 80% and back) of an ROI in the excretory cell of L4 transgenic animals of the genotype: *catp-5(tm4481)* + *P*_*pgp-12*_*catp-5*::*gfp*_*let-858*_ + *rol-6(su1006)*, *bgIs312[pes-6*::*gfp]*, *xnIs459[yfp*::*ral-1a + unc-119(+)]* III and *xnIs484 [mCherry*::*sec-10 + unc-119(+)]*. Fluorescence recovery over time was measured via Fiji/imageJ 2.0.0 [36]. In order to compensate for any movement, we averaged 4–10 pixel of unbleached areas left and right to the ROI and used these measurements to normalize according to [37]. Statistical analysis was performed by using RStudio Version 1.0.143 (https://www.rstudio.com). Shapiro-Wilk test in combination with graphical analysis was used to test for normality. Equality of variance was tested via Levene's test. We used an ANOVA (command: aov) and subsequent Tukey post-hoc test.

### Co-localization

Head neurons: Animals were immobilized with 1 mM sodium azide. Animals were mounted on 4% agarose pads. Spermatheca: Gonads were dissected in L-15/FBS medium [38] with 20 gauge hypodermic needles and mounted without any agar pad directly on microscope slides. Leica TCS SP5 II confocal microscope was used for imaging.

### Brood size estimation

Triplicates of 10 L4 animals for each genotype were transferred to freshly seeded NGM plates (at 20°C). To obtain the complete progeny of all 10 animals per triplicate, worms were transferred to new seeded NGM plates twice a day (morning and afternoon). Once the progeny on a plate reached young adult stage the plates were shifted to 10°C. After six days, the complete progeny were washed off with MPEG (M9 plus 0.05% polyethylene glycol 8000 (PEG)) thereafter frozen at -80°C. Water plus PEG (0.05%) was added to a volume of 30 ml. After shaking, 3 ml of worm suspension was taken to count the average brood size of 1 animal. Statistical analysis was performed by using RStudio Version 1.0.143 (https://www.rstudio.com). Shapiro-Wilk test in combination with graphical analysis was used to test for normality. Equality of variance was tested via Levene's test. We used an ANOVA (command: aov) and subsequent Tukey post-hoc test.

### Growth rate estimation

Triplicates of 10 adult animals for each genotype were transferred to freshly seeded NGM plates (at 20°C). All 10 animals were removed after exactly 1 h. 48 h later, the progeny were scored according their developmental stage. The progeny of all 3 replicates were pooled for analysis. Statistical analysis was performed by using RStudio Version 1.0.143 (https://www.rstudio.com). We used Fisher’s exact test and subsequent Holm’s correction for multiple comparison (command: pairwiseNominalIndependence).

## Results

### *C*. *elegans* P5B isoforms and sequence similarity

According to WormBase (WS262), the *catp-5* locus gives rise to four mRNAs, which differ only in their 5' end regions (Fig 2A). The *catp-5a*.*1* and *catp-5a*.*2* transcripts both encode the same protein isoform, CATP-5a (1174 aa). *catp-5b* encodes CATP-5b (1203 aa) and *catp-5c* encodes CATP-5c (1202 aa). All three isoforms display the typical P5B ATPase structure: One membrane associated segment (Ma) [9], ten transmembrane segments (M1-M10), an actuator domain formed by two cytoplasmic loops (between Ma-M1 and M2-M3), plus a nucleotide binding domain and a phosphorylation domain located in the large cytoplasmic loop (M4-M5, Fig 1, Fig 2B).

The *catp-6* locus produces three different mRNAs (WS262), each with a different 5' end region and encoding a distinct protein isoform. *catp-6a* and *catp-6c* encode isoforms that display the typical P5B ATPase structure (CATP-6a, 1256 aa, and CATP-6c 1207 aa). However, the isoform encoded by *catp-6b* is only 893 aa in length and lacks residues N-terminal to M3. We do not know whether CATP-6b is expressed and/or functionally significant in vivo; however, the RNA seq data summarized on WormBase (WS262), suggest that *catp-6b* is not a highly-abundant transcript.

The *catp-7* locus gives rise to three different mRNAs (WS262), each with a different 5' end region. *catp-7a*.*1* and *catp-7a*.*2* each encode CATP-7a, 1127 aa. *catp-7b* encodes CATP-7b, 1111 aa. CATP-7a and CATP-7b both include all of the canonical P-type ATPase domains.

We compared the core amino acid sequences of *C*. *elegans* CATP-5, CATP-6 and CATP-7 according to [7]. Based on this, we found that CATP-6 is slightly more similar to CATP-7 than it is to CATP-5 (78.24% vs 75.42% identity) (Fig 2C), whereas CATP-5 and CATP-7 are the least similar, sharing 70.0% identity.

### Phylogenetic analysis of paralogous P5B ATPases

**Fig 3:**
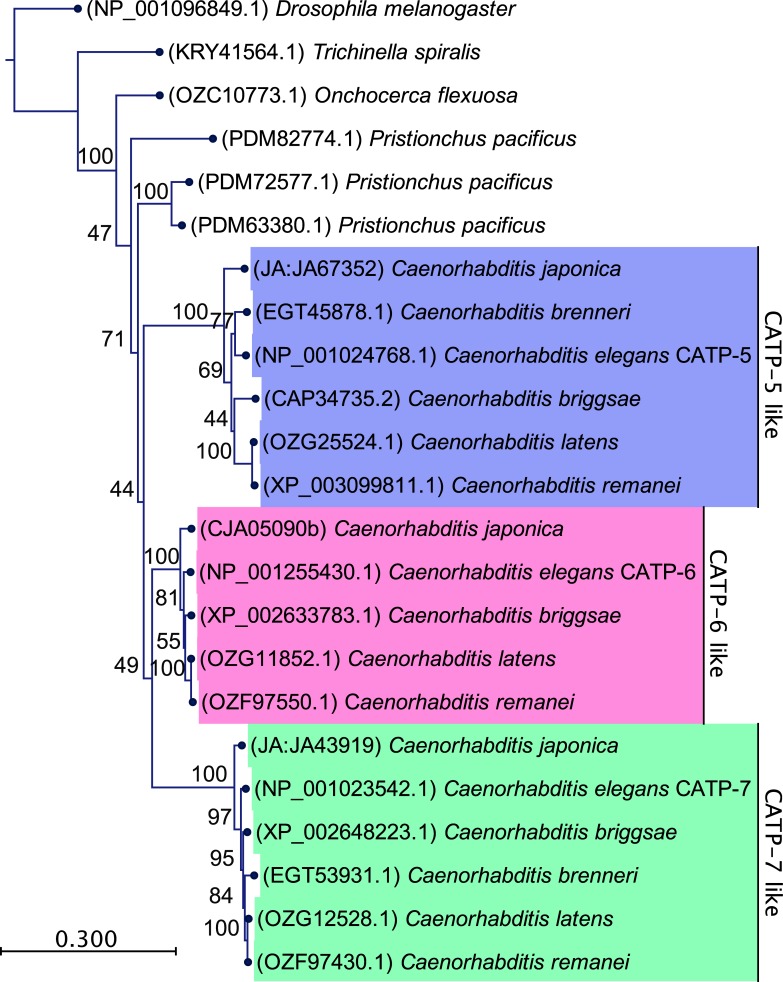
Phylogenetic analysis of paralogous P5B ATPases. Maximum Likelihood tree of 9 nematode species (including 6 Caenorhabditis species) and *Drosophila melanogaster* as an outgroup. Statistical model: LG+G. Bootstrap values for each node are indicated (100 replicates).

We obtained orthologous P5B ATPase aa sequences of five additional Caenorhabditis species (*C*. *japonica*, *C*. *brenneri*, *C*. *briggsae*, *C*. *latens* and *C*. *remanei*), more distant nematode species (*Pristionchus pacificus*, *Onchocerca flexuosa* and *Trichinella spiralis*) and *Drosophila melanogaster* as an outgroup from http://www.wormbase.org, http://www.flybase.org and from https://www.ncbi.nlm.nih.gov. Since, the genomes of these species are thought to be fully sequenced [39–42], it is very likely that we have included all paralogous P5B ATPases for each species. We constructed a Maximum Likelihood tree by using the model LG+G [22] (Fig 3). We found that each Caenorhabditis species, except *C*. *brenneri*, has one CATP-5-like, one CATP-6-like and one CATP-7-like P5B ATPase. The CATP-6-like and CATP-7-like paralogs are more similar to each other then they are to the CATP-5-like paralogs, suggesting that *catp-6* and *catp-7* originated from the most recent gene duplication event. Although the genome of *Pristionchus pacificus* also codes for three paralogous P5B ATPases, these do not group with the paralogous P5B ATPases of the Caenorhabditis clade. The genomes of more distantly related nematodes, such as *Onchocerca flexuosa* and *Trichinella spiralis*, encode only a sole P5B ATPase.

### CATP-5 expression pattern

In previous studies, the spatiotemporal expression patterns of CATP-5 and CATP-6 were characterized by the expression of tagged proteins using extrachromosomal arrays [11,12]. In the current study, we sought to verify and extend these findings by using both extrachromosomal arrays and by tagging the endogenous loci using CRISPR/Cas9.

**Fig 4:**
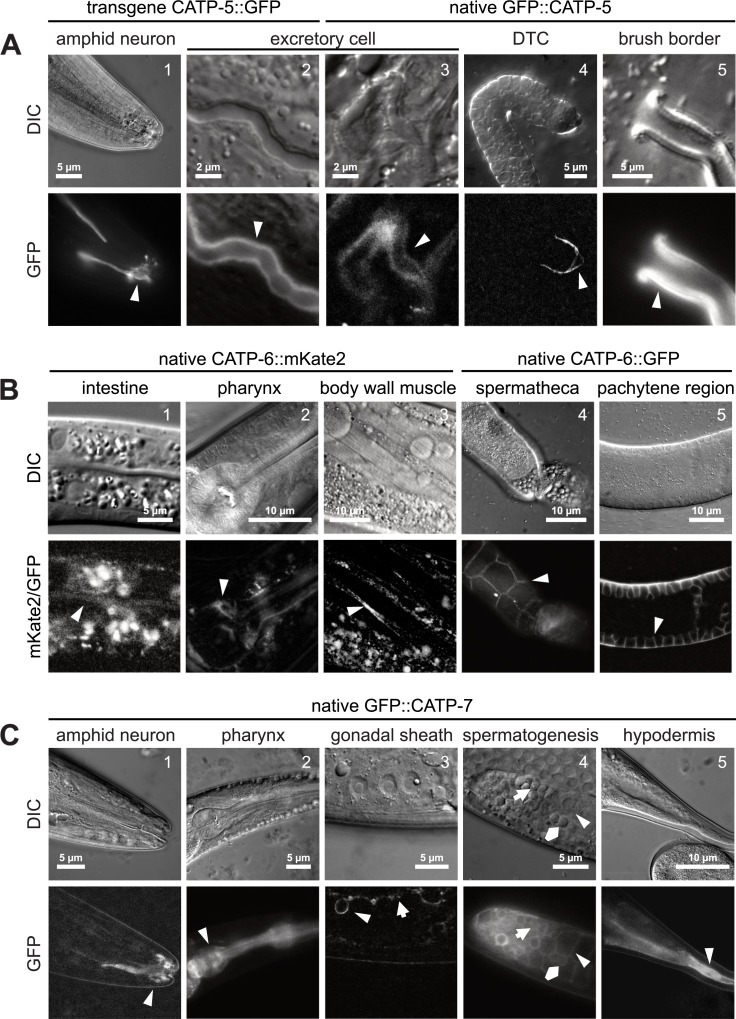
Tissue-specific expression patterns of CATP-5, CATP-6 and CATP-7. Transgene CATP-X::FP indicates expression from an extrachromosomal array. Native CATP-X::FP indicates CRISPR/Cas9 tagged endogenous locus. (A) Representative tissues in which CATP-5 is expressed. *Ex [P*_*catp-5*_*catp-5*::*gfp;rol-6(d)]* and *catp-5(dx187[gfp*::*catp-5 + loxP])* X. (B) Representative tissues in which CATP-6 is expressed. *catp-6(dx183[catp-6*::*gfp*::*3xFlag + loxP])* IV and *catp-6(dx179[catp-6*::*degron*::*mKate2*::*3xFlag + loxP])* IV. (C) Representative tissues in which CATP-7 is expressed. *catp-7(dx185[gfp*::*catp-7 + loxP])* IV. Arrowheads indicate fluorescence signal (B1 and B3 GFP panels contain extensive autofluorescence signal from intestine). (C.3) Arrowhead: Sheath cell with an engulfed an apoptotic germ cell. Arrow: Sheath cell. (C.4) Arrowhead: Spermatocyte. Arrow: Spermatid. Pentagram: Residual body. (C.5) Arrowhead: Hyp10.

In order to generate a tagged version of CATP-5 for use on extrachromosomal arrays, we fused the coding sequence of *gfp* to the 3’ end of the *catp-5* coding sequence, and drove expression using the putative *catp-5* promoter (4.1 kb upstream of *catp-5a*.*1*). Consistent with the report of Heinick et al. [11], we found that CATP-5::GFP is expressed in the intestinal cells, where it localizes to the apical brush border (Fig 4A.5), and the excretory cell, where it appears to be distributed throughout the cytosol (Fig 4A.2,3). In addition, we observed CATP-5::GFP expression in the spermatheca (localized to the apical face, see below) and amphid sensory neurons (localized to the sensilla) (Fig 4A.1).

Since the presence of a C-terminal GFP tag could potentially interfere with proper subcellular localization, we used CRISPR/Cas9-mediated recombination to tag the endogenous CATP-5 protein at the N-terminus of CATP-5a (Fig 1) [31]. We refer to the protein encoded by the tagged locus (*catp-5(dx187[gfp*::*catp-5 + loxP])* X), as native GFP::CATP-5. The expression pattern of native GFP::CATP-5 is nearly identical to that of CATP-5::GFP. However, we also detected expression on the plasma membrane of the distal tip cells (DTC) (Fig 4A.4) and on the plasma membrane of pachytene-stage germ cells (see below). We also generated a native *catp-5* allele in which *gfp* is inserted at the N-terminus of CATP-5c; however, we did not observe any GFP signal in these animals.

### CATP-6 expression pattern

To create a red fluorescent protein tagged version of CATP-6 for use on extrachromosomal arrays, we fused the coding sequence for mKate2 to the 3’ end of the *catp-6* coding sequence, with expression driven by the putative *catp-6* promoter (1135 bp 5’ of exon 1 of *catp-6a*). The expression pattern of this transgene was essentially identical to that of the *gfp*-modified version of fosmid WRM067B_F08 used in our previous study [12]. However, we did observe that the signal from CATP-6::mKate was more finely resolved than that of CATP-6::GFP.

To obtain tagged native versions of CATP-6, we used CRISPR/Cas9 to produce two different tagged versions of *catp-6*, each with a fluorescent protein (FP) fused to the end of the coding sequence (Fig 1) [32]. One allele (*catp-6(dx183[catp-6*::*gfp*::*3xFlag + loxP])* IV) utilized GFP, whereas the other (*catp-6(dx179[catp-6*::*degron*::*mKate2*::*3xFlag + loxP])* IV) utilized mKate2 tag preceded by a degron motif [33]. (The degron segment is not functional in this genetic background). The expression patterns of the native FP-fusions are very similar to those observed using extrachromosomal transgenes. CATP-6::FP is expressed in a punctate pattern in many neurons in the head and tail, all body wall muscles, the posterior gut, the spermatheca, the DTC and the pharyngeal cells (Fig 4B and below). CATP-6::FP is also expressed in the gonadal sheath cells, where it appears to be closely associated with the plasma membrane and is not punctate (see below). Notably, native CATP-6::FP is strongly expressed in the germ line within the mitotic region, the transition zone and the pachytene region (Fig 4B.5 and below), and appears to localize to the plasma membrane.

### CATP-7 expression

To construct a tagged version of CATP-7 for use on extrachromosomal arrays, we inserted *gfp* at the 3' end of the *catp-7* coding sequence, with expression driven by the putative *catp-7* promoter (2377 bp 5’ of exon1 of *catp-7a*). To tag the native locus, we inserted the coding sequence of either *gfp* or *mcherry* into the second exon of isoform *catp-7a* via CRISPR/Cas9 (*catp-7(dx185[gfp*::*catp-7 + loxP])* IV and *catp-7(dx191[mcherry*::*catp-7 + loxP])* IV), Fig 1, [31]). The expression and localization of the native N-terminal fusion GFP::CATP-7 closely resemble those of the C-terminally tagged extrachromosomal transgene. Native GFP::CATP-7 is expressed closely associated with the plasma membrane of the amphid sensory neurons (localized to the sensilla), the gonadal sheath cells, the spermatheca, the hypodermis and the excretory cell (Fig 4C and below). GFP::CATP-7 is expressed in most pharyngeal cells, where it localizes to the plasma membrane, plus internal membranous tubules (Fig 4C.2). GFP::CATP-7 is also expressed in the intestine, where it is associated with tubular structures in the basolateral domain and with vesicles in the apical domain immediately below the microvilli (see below; basolateral not shown). GFP::CATP-7 is strongly expressed in spermatocytes and spermatids (Fig 4C.4 and below). In the case of mCherry::CATP-7, we observed that the tagged protein was expressed and localized as for GFP::CATP-7, but it also accumulated in large aggregates within the cytosol (see below). This is most likely due to multimerization of mCherry, since this has been reported by others [43].

The CRISPR/Cas9-mediated single *fp*-fusion strains were not backcrossed after they were generated, but they did not show any obvious differences compared to wt N2. We estimated the average brood size for each single *gfp*-fusion strain in comparison with wt N2 (Table 1). There is no statistical difference detectable when the brood size of each CRISPR engineered strain is compared to N2. But the brood size of native *catp-6*::*gfp* differs from the brood size of native *gfp*::*catp-5* (Table 1, group b and a, ANOVA, Tukey post-hoc test, adj. *p*-value = 0.02327153).

**Table 1:** Estimated average brood size. Mean of 3 replicates. SEM = Standard error of the mean. Group letter: Groups that share the same letter (ab) do not statistically differ from each other. Groups with different letters are significantly different from each other (a, b and c). ANOVA, Tukey post-hoc test. Adj. *p*-value threshold < 0.05.

Genotype	Mean	SEM [±]	Group letter *p*-value < 0.05
N2	317	5	ab
*catp-5(tm4481)* X	303	10	ab
*catp-6(ok3473)* IV	95	7.3	c
*catp-7(tm4438)* IV	295	9.9	ab
*catp-5(dx187[gfp*::*catp-5 + loxP])* X	281	10.6	b
*catp-6(dx183[catp-6*::*gfp*::*3xFlag + loxP])* IV	370	41.8	a
*catp-7(dx185[gfp*::*catp-7 + loxP])* IV	351	11.9	ab
*catp-6(dx179[catp-6*::*degron*::*mKate2*::*3xFlag + loxP])* IV; *catp-5(dx187[gfp*::*catp-5 + loxP])* X	314	5.7	ab
*catp-6(dx179[catp-6*::*degron*::*mKate2*::*3xFlag + loxP])* *catp-7(dx193[gfp*::*catp-7 + loxP])* IV	299	5.5	ab

Additionally, we estimated the growth rate of the progeny (1 h layoff) from 30 worms (Table 2). 48 h after the layoff, 98–100% of the progeny of the single *fp*-fusion strain had reached the L4 stage, which is comparable to wt N2.

**Table 2:** Estimated growth rate. The progeny (1 h layoff) from 30 worms for each genotype were scored after 48 h according to their developmental stage. L3 = Larval stage 3. L4 = Larval stage 4. Both presented as percentage. n = Total number of scored worms. Groups that share the same letter (x) do not statistically differ from each other. Groups with different letters are significantly different from each other (x and y). Fisher's exact test. Adj. *p*-value threshold < 0.05.

Genotype	L3 [%]	L4 [%]	n	Group letter *p*-value < 0.05
N2	1	99	233	x
*catp-5(tm4481)* X	1	99	276	x
*catp-6(ok3473)* IV	38	63	40	y
*catp-7(tm4438)* IV	1	99	278	x
*catp-5(dx187[gfp*::*catp-5 + loxP])* X	1	99	314	x
*catp-6(dx183[catp-6*::*gfp*::*3xFlag + loxP])* IV	0	100	219	x
*catp-7(dx185[gfp*::*catp-7 + loxP])* IV	2	98	224	x
*catp-6(dx179[catp-6*::*degron*::*mKate2*::*3xFlag + loxP])* IV; *catp-5(dx187[gfp*::*catp-5 + loxP])* X	1	99	283	x
*catp-6(dx179[catp-6*::*degron*::*mKate2*::*3xFlag + loxP])* *catp-7(dx193[gfp*::*catp-7 + loxP])* IV	0	100	201	x

### P5B subcellular localization in selected tissues

#### P5B expression in intestinal cells

**Fig 5:**
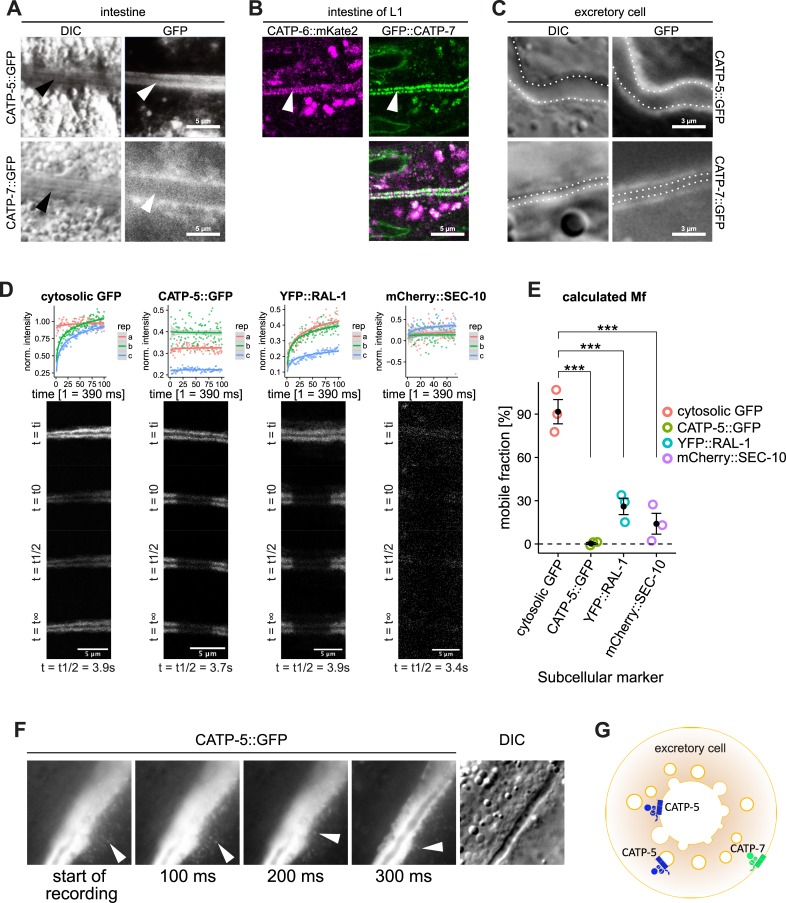
P5B expression in intestinal cells and the excretory cell. (A) Expression of transgene CATP-5::GFP vs. transgene CATP-7::GFP in intestinal cells. *Ex [P*_*catp-5*_*catp-5*::*gfp;rol-6(d)]* and *Ex [P*_*catp-7*_*catp-7*::*gfp;rol-6(d)]*. Arrowheads indicate apical brush border. (B) Expression of native CATP-6:;mKate2 vs. native GFP::CATP-7 in intestinal cells of L1. *catp-6(dx183[catp-6*::*gfp*::*3xFlag + loxP])* IV and *catp-6(dx179[catp-6*::*degron*::*mKate2*::*3xFlag + loxP])* IV. (C) Expression of transgene CATP-5::GFP vs. transgene CATP-5::GFP in the excretory cell. *Ex [P*_*catp-5*_*catp-5*::*gfp;rol-6(d)]* and *Ex [P*_*catp-7*_*catp-7*::*gfp;rol-6(d)]* (D) FRAP experiment (60 s bleaching) of transgene CATP-5::GFP vs. cytosolic GFP, YFP::RAL-1 and mCherry::SEC-10. *catp-5(tm4481)*; *Ex [P*_*pgp-12*_*catp-5*::*gfp;rol-6(d)]*, *bgIs312[pes-6*::*gfp]*, *xnIs459[yfp*::*ral-1a + unc-119(+)]* III and *xnIs484 [mCherry*::*sec-10 + unc-119(+)]*. (E) Calculated mobile fraction (Mf) of FRAP experiment. ANOVA, Tukey post-hoc test, adj. *p*-value: *** ≤ 0.001. (F) CATP-5::GFP positive vesicles (arrowheads) escape from ruptured excretory cell. (G) Schematic overview of CATP-5::GFP and CATP-7::GFP localization in the excretory cell.

CATP-5 and CATP-7 are both expressed in the intestine. Native GFP::CATP-5 and transgene CATP-5::GFP localize to the apical brush border of the intestinal cells (Fig 4A, Fig 5A). Native GFP::CATP-7 and transgene CATP-7::GFP localize to small vesicles immediately beneath the apical brush border (Fig 5A). We also observed that both GFP::CATP-7 and CATP-6::GFP localize to tubular structures in the basolateral domain of the intestinal cells. In first stage larvae, native GFP::CATP-7 and native CATP-6::mKate2 localize to the apical brush border of the intestinal cells (Fig 4B, Fig 5B).

#### P5B expression in the excretory cell

CATP-7::GFP is clearly localized to the basolateral membrane of the excretory cell, since the GFP signal is not in close proximity to the lumen of the excretory canal on DIC (Fig 5C and 5G). On the other hand, CATP-5::GFP signal appears distributed throughout the cytosol of the excretory cell. We wondered whether this localization might be an artifact due to soluble GFP derived from the transgene (e.g., via proteolytic cleavage, or cryptic promoter usage). To test this hypothesis, we performed a Fluorescence Recovery after Photobleaching (FRAP) experiment [37]. We photobleached FP-tagged proteins in the excretory cell and measured the signal-recovery over time, normalized to an unbleached area of the cell (Fig 5D). We tested CATP-5::GFP^excretory^ specifically expressed in the excretory cell of *catp-5* mutant animals (*catp-5(tm4481)* + *P*_*pgp-12*_*catp-5*::*gfp* + *rol-6(su1006)*) and cytosolic GFP (*bgIs312[pes-6*::*gfp]*) [18], plus two membrane-associated controls: YFP::RAL-1 (*xnIs459[yfp*::*ral-1a]*, marker for cytosolic vesicles) and mCherry::SEC-10 (*xnIs484[mCherry*::*sec-10]*, marker for the luminal membrane [15]). To quantitate mobility, we calculated the mobile fraction (Mf) of each FP-fusion (Fig 5E).

As expected, cytosolic GFP shows a calculated Mf of 92% (Fig 5E). Whereas CATP-5::GFP^excretory^ displays a Mf of only 0.33%, which is highly significantly different from cytosolic GFP (ANOVA, Tukey post-hoc test, adj. *p*-value = 0.0000307). Thus, it is very unlikely that the signal we observe is coming from cytosolic GFP in the excretory cell for transgene *catp-5*::*gfp*^*excretory*^.

The cytosolic vesicle-associated control YFP::RAL1 shows a Mf of 26% and the luminal membrane control SEC10::mCherry shows a Mf of 14%. Both Mf values are significantly different from the Mf value of cytosolic GFP (Fig 5E, ANOVA, Tukey post-hoc test, adj. *p*-value = 0.0003369 and adj. *p*-value = 0.0001015 respectively). But there is no statistical differences detectable between the Mfs of: CATP-5::GFP^excretory^ vs. YFP::RAL1, CATP-5::GFP^excretory^ vs. mCherry::SEC-10 nor YFP::RAL1 vs. mCherry::SEC-10.

During one imaging experiment, we serendipitously ruptured the basolateral membrane of the excretory cell and could thus observe consequences of cytoplasmic leakage into the extracellular fluid. In this case, we observed that CATP-5::GFP escaped from the cell in the form of small GFP-positive punctae, consistent with vesicular localization (Fig 5F). In addition, as cytosolic signal became depleted, it became possible to see that some of the CATP-5::GFP was tightly associated with the luminal membrane. We were able to reproduce the observation of persistent luminal association of CATP-5::GFP in two other experiments in which we used pressure on the coverglass to rupture the worm and sever the excretory canal.

#### Subcellular localization of CATP-6 in neurons and pharynx

In order to determine the subcellular compartment to which CATP-6 localizes, we imaged CATP-6::FP in relation to various compartment markers in head neurons via confocal microscopy.

**Fig 6:**
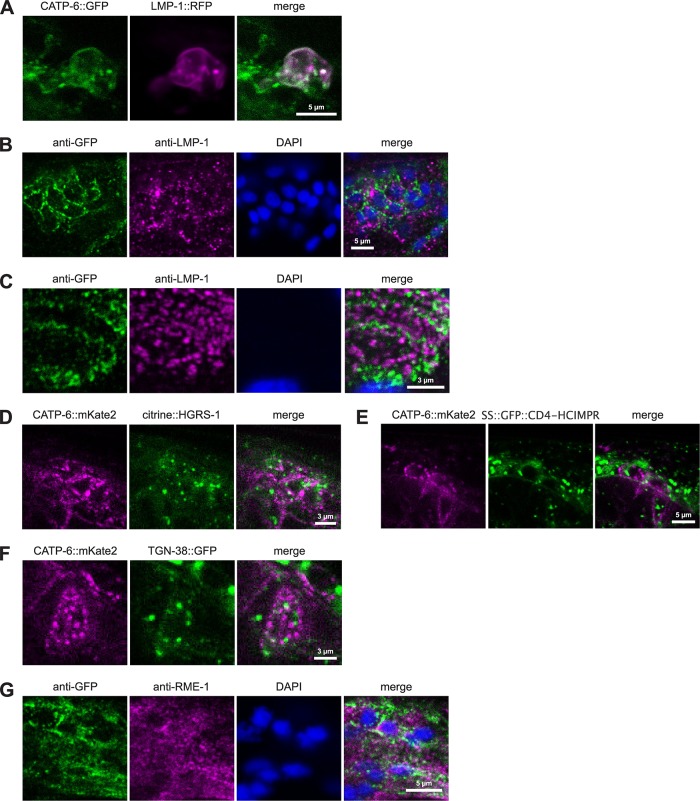
Subcellular localization of CATP-6 in neurons and pharynx. (A) Co-localization of native CATP-6::GFP vs. LMP-1::RFP in living neurons. *catp-6(dx183[catp-6*::*gfp*::*3xFlag + loxP])* IV, *Ex [P*_*catp-6*_*catp-6*::*gfp;P*_*sth-1*_*lmp-1*::*rfp;rol-6(d)]*. (B-C) Immunostaining of native CATP-6::GFP vs. native LMP-1 in neurons and pharyngeal bulb. *catp-6(dx183[catp-6*::*gfp*::*3xFlag + loxP])* IV. (D-F) Co-localization of native CATP-6::mKate2 vs. citrine::HGRS1, SS::GFP::CD4-HCIMPR, and TGN-38::GFP in living neurons. *catp-6(dx179[catp-6*::*degron*::*mKate2*::*3xFlag + loxP])* IV, *pwIS1039[p*_*snx-1*_*citrine*::*hgrs-1*_*unc-54*_ *UTR-cb-unc-119]*, *pwIs1176[p*_*snx-1*_*ss-gfp-cd4-hcimpr*_*unc-54*_ *UTR-cb-unc-119]; unc-119(ed3)* and *pwIs1175[p*_*snx-1*_*tgn-38*::*gfp*_*unc-54*_ *UTR-cb-unc-119]; unc-119(ed3)*. (G) Immunostaining of native CATP-6::GFP vs. native RME-1 in neurons. *catp-6(dx183[catp-6*::*gfp*::*3xFlag + loxP])* IV.

When we expressed the lysosomal marker, LMP-1::RFP in neurons using the *rgs-1* promoter, we found strong colocalization with CATP-6::GFP. However, LMP-1::RFP and CATP-6::GFP both localized to the plasma membrane in these cells (Fig 6A). Evidently, overexpression of LMP-1 and/or the presence of the C-terminal RFP tag leads to inappropriate localization of both LMP-1 and CATP-6. As an alternative approach, we performed immunocytochemistry by staining for native CATP-6::GFP (anti-GFP) and endogenous LMP-1 (anti-LMP-1, Fig 6B and 6C). With this method, we did not observe strong colocalization of LMP-1 and CATP-6::GFP, either within neurons or in pharyngeal cells (Fig 6B and 6C).

We used transgenic strains generated by Norris et al. [19] to test whether CATP-6::FP colocalizes with either of two endosomal markers, citrine::HGRS-1 or SS::GFP::CD4-HCIMPR. Although each of these markers exhibits a punctate subcellular localization pattern, they are clearly distinct from CATP-6::mKate2. (Fig 6D and 6E). We also examined the trans-Golgi marker, TGN-38::GFP [19], and found that although it does exhibit a punctate subcellular localization, this is also distinct from CATP-6::mKate2 (Fig 6F).

As a marker for recycling endosomes we used immunostaining to assess colocalization of native CATP-6::GFP and RME-1 in neurons (Fig 6G). Again, although the pattern of RME-1 appears punctate, it is largely distinct from that of CATP-6::GFP.

#### Subcellular localization of CATP-6 in the spermatheca

In the spermathecal epithelial cells, CATP-6::FP localizes to cytoplasmic punctae that probably correspond to membranous vesicles near the basolateral plasma membrane. We analyzed native CATP-6::GFP in combination with transgene CATP-6::GFP (*gfp* modified fosmid WRM067B_F08 to increase GFP signal) in relation to RFP-labeled compartment markers expressed in the spermatheca (using the *sth-1* promoter [26]).

**Fig 7:**
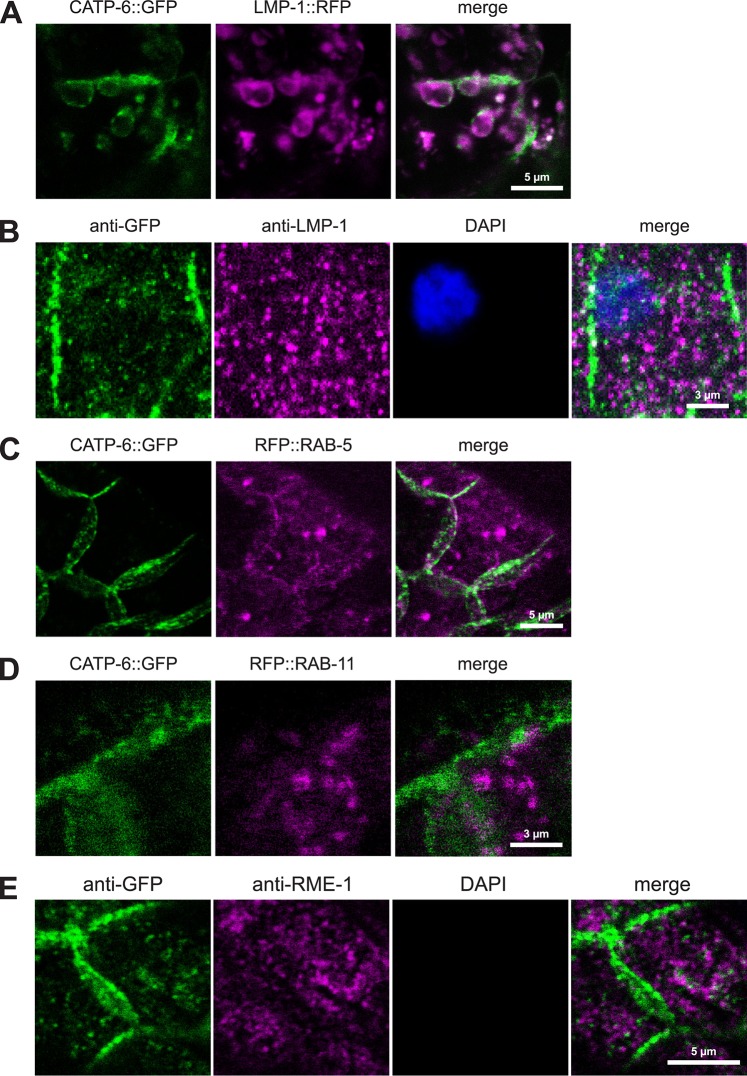
Subcellular localization of CATP-6 in the spermatheca. (A) Co-localization of native CATP-6::GFP vs. LMP-1::RFP in living spermatheca. *catp-6(dx183[catp-6*::*gfp*::*3xFlag + loxP])* IV, *Ex [P*_*catp-6*_*catp-6*::*gfp;P*_*sth-1*_*lmp-1*::*rfp;rol-6(d)]*. (B) Immunostaining of native CATP-6::GFP vs. native LMP-1 in the spermatheca. *catp-6(dx183[catp-6*::*gfp*::*3xFlag + loxP])* IV. (C-D) Co-localization of native CATP-6::GFP vs. RFP::RAB-5 and RFP::RAB-11 in living spermatheca. *catp-6(dx183[catp-6*::*gfp*::*3xFlag + loxP])* IV, *Ex [P*_*catp-6*_*catp-6*::*gfp;P*_*sth-1*_*rfp*::*rab-5;rol-6(d)]* or *Ex [P*_*catp-6*_*catp-6*::*gfp;P*_*sth-1*_*rfp*::*rab-11;rol-6(d)]*. (E) Immunostaining of native CATP-6::GFP vs. native RME-1 in the spermatheca. *catp-6(dx183[catp-6*::*gfp*::*3xFlag + loxP])* IV.

In living spermathecal cells, CATP-6::GFP strongly colocalizes with LMP-1::RFP expressed from a transgene (Fig 7A). However, the CATP-6::GFP positive vesicles are much larger than usual, so we suspected that the colocalization might be an artifact. Therefore, we used immunostaining to assess colocalization of native CATP-6::GFP and LMP-1 within the spermatheca (Fig 7B). In these preparations, the LMP-1 and CATP-6::GFP localization patterns are clearly different. LMP-1 is not associated with the basolateral cell membranes and the CATP-6 positive vesicles are relatively normal in size and shape.

In the case of RFP::RAB-5 we observed overlap with CATP-6::GFP in proximity to the plasma membrane of basolateral cell-cell junctions. This is consistent with the expected localization of RAB-5 to early endosomes (Fig 7C). The localization pattern of RFP::RAB-11 (expected to label recycling endosomes), is largely distinct from that of CATP-6::GFP (Fig 7D). We used immunostaining to assess colocalization of native CATP-6::GFP and RME-1 as a second marker for recycling endosomes within the spermatheca (Fig 7E). Again, the pattern of RME-1 is largely distinct from that of CATP-6::GFP.

### Overlapping expression patterns in developing reproductive tissues

**Fig 8:**
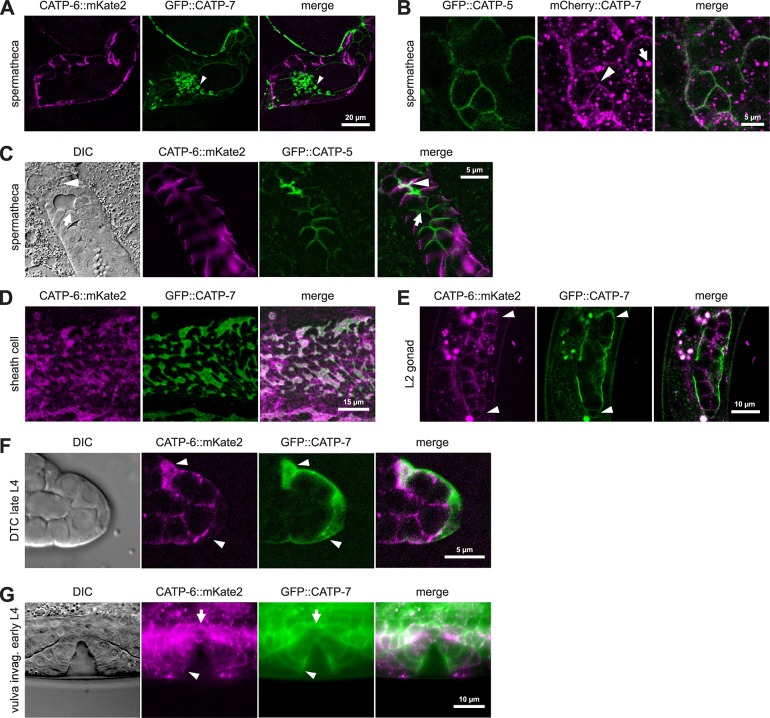
Overlapping expression patterns in developing reproductive tissues. (A) Native CATP-6::mKate2 vs. native GFP::CATP-7 in living spermatheca. *catp-6(dx179[catp-6*::*degron*::*mKate2*::*3xFlag + loxP]) catp-7(dx193[gfp*::*catp-7 + loxP])* IV. (B) Native GFP::CATP-5 vs. native mCherry::CATP-7 in living spermatheca. *catp-7(dx191[mcherry*::*catp-7 + loxP])* IV; *catp-5(dx187[gfp*::*catp-5 + loxP])* X. Arrowhead: Membrane bound mCherry signal. Arrow: mCherry aggregates. (C) Native CATP-6::mKate2 vs. native GFP::CATP-5 in living spermatheca. *catp-6(dx179[catp-6*::*degron*::*mKate2*::*3xFlag + loxP])* IV; *catp-5(dx187[gfp*::*catp-5 + loxP])* X. Arrowhead: Apical luminal membrane. Arrow: Apical lateral borders. (D-G) Native CATP-6::mKate2 vs. native GFP::CATP-7 in living sheath cell (adult) and developing gonad (L2), DTC (late L4) and vulva morphogenesis (early L4). *catp-6(dx179[catp-6*::*degron*::*mKate2*::*3xFlag + loxP]) catp-7(dx193[gfp*::*catp-7 + loxP])* IV. Arrowheads: (E and F): DTCs. Note: the DTCs extend long processes to cover nearby germ cells, even in early larvae (G): Colocalization of CATP-6::mKate2 and GFP in central somatic gonad (arrow) and developing vulva (arrowhead).

All three paralogous P5B ATPases are expressed in the spermathecal epithelial cells. In order to directly compare their localization patterns, we constructed strains in which different pairs of proteins are tagged. Since the *catp-6* and *catp-7* loci are very tightly linked on chromosome IV, it was not possible to obtain a homozygous double-labeled strain by meiotic recombination. Therefore, we used CRISPR/Cas9 to generate a strain that expresses both native CATP-6::mKate2 and native GFP::CATP-7. The strain was not backcrossed but did not show any obvious difference compared to wt N2. We estimated the average brood size of this strain (Table 1) plus the growth rate of the progeny (1 h layoff) (Table 2) and did not observe any difference compared to wt N2. In double-tagged animals, it is clear that the localization patterns of the two proteins within the spermathecal cells are distinct: CATP-6::mKate2 localizes to punctae in the basolateral domain, whereas GFP::CATP-7 localizes to the lateral and luminal cell borders, as well as to spermatids (Fig 8A).

By intercrossing, we obtained a strain that expresses both native CATP-6::mKate2 and native GFP::CATP-5 (Fig 8C). This double-tagged strain did not differ from wt N2 in terms of brood size or growth rate (Table 1, Table 2). Native GFP::CATP-5 is expressed in a pattern qualitatively similar to that of native GFP::CATP-7, localizing to the apical lateral and luminal borders of the spermatheca (compare Fig 8A to Fig 8C), but GFP::CATP-5 is not detectable in spermatids. We obtained a strain that expresses both native mCherry::CATP-7 and native GFP::CATP-5 via crossing of the single *fp*-tagged strains (Fig 8B). Membrane-associated mCherry::CATP-7 signal localizes to the same lateral and luminal borders as GFP::CATP-5, confirming that both CATP-5 and CATP-7 localize to the same subcellular compartment in the spermatheca (Fig 8B).

In the gonadal sheath cells, native GFP::CATP-7 localizes to large, raft-like patches on the plasma membrane, where it colocalizes with native CATP-6::mKate2 (Fig 8D). Moreover, we observe areas of the sheath cell that are positive for native CATP-6::mKate2, but not for native GFP::CATP-7 (Fig 8D). In favorable cross-section images, we were able to observe that native GFP::CATP-7 and native CATP-6::mKate2 are present on both the outer (pseudocoelom-facing) inner (germline-facing) plasma membrane surfaces of the sheath cell (see below).

Native GFP::CATP-7, as well as CATP-6::mKate2 are expressed in the DTCs of early stage gonads (L2, Fig 8E). CATP-6::mKate2 is also expressed in larval germ cells, exhibiting a puncate localization pattern, consistent with endosomal localization. Native GFP::CATP-7 and native CATP-6::mKate2 are also both expressed in the DTCs of L4 animals, although the CATP-6 expression is comparatively weak (Fig 8F). As in other tissues, GFP::CATP-7 and GFP::CATP-5 localization to the plasma membrane appears fairly smooth/continuous, whereas CATP-6::mKate2 appears punctate, consistent with endosomal localization (Fig 4A.4, Fig 8E and 8F).

In addition to somatic gonad cells, native GFP::CATP-7 and CATP-6::mKate2 are both strongly expressed in the developing vulva (Fig 8G). Again, GFP::CATP-7 appears continuously associated with the plasma membrane, whereas CATP-6::mKate2 localization is punctate.

### Overlapping expression in the germ line

**Fig 9:**
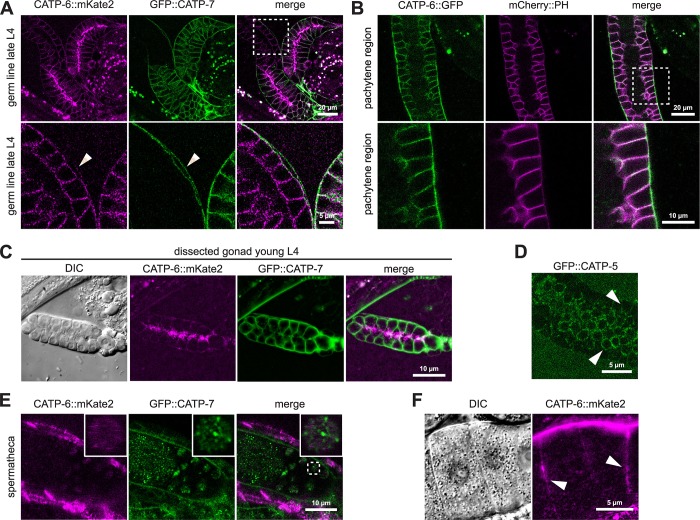
Overlapping expression in the germ line. (A, C and E) Native CATP-6::mKate2 vs. native GFP::CATP-7 in germ line during spermatogenesis (L4, A and C) and in spermatheca of young adults (E). *catp-6(dx179[catp-6*::*degron*::*mKate2*::*3xFlag + loxP]) catp-7(dx193[gfp*::*catp-7 + loxP])* IV. (B) Co-localization of native CATP-6 vs. mCherry PH. *catp-6(dx183[catp-6*::*gfp*::*3xFlag + loxP])* IV; *ltIs44(P*_*pie-1*_*mCherry*::*ph*^*PLCδ*^*)* V. (D) Native GFP::CATP-5 in pachytene region of adults. *catp-5(dx187[gfp*::*catp-5 + loxP])* X. (F) Native CATP-6::mKate2 localizes to the plasma membrane of diakinesis stage oocytes (arrowhead); cytoplasmic signal is autofluorescence. *catp-6(dx179[catp-6*::*degron*::*mKate2*::*3xFlag + loxP])*.

Native CATP-6::FP is strongly expressed in the adult germ line, and colocalizes with the plasma membrane marker *ltIs44(P*_*pie-1*_*mCherry*::*ph*^*PLCδ*^*)* within the mitotic, transition, and pachytene regions (Fig 9B). CATP-6::mKate2 is also associated with the plasma membrane of diakinesis-stage oocytes (Fig 9F). In L4 stage hermaphrodites, CATP-6::mKate2 localizes to vesicles in the rachis between the spermatocytes during spermatogenesis (Fig 9A and 9C). We also detect a weak cytoplasmic signal for CATP-6::mKate2 in spermatids (Fig 9E). Native GFP::CATP-7 is not expressed in the mitotic, transition or pachytene regions of the adult hermaphrodite germ line; however, it is strongly associated with the plasma membrane of spermatocytes and residual bodies in L4 larvae and young adults (Fig 9A and 9C, Fig 4C.4). In spermatids, GFP-CATP-7 localizes to punctae that probably correspond to fibrous body-membranous organelles (FB-MOs [44], Fig 9E). Native GFP::CATP-5 is expressed very weakly in the mitotic, transition and pachytene region of the germ line and also localizes to the plasma membrane (Fig 9D).

### Phenotypic analysis of single, double and triple null mutants

**Fig 10:**
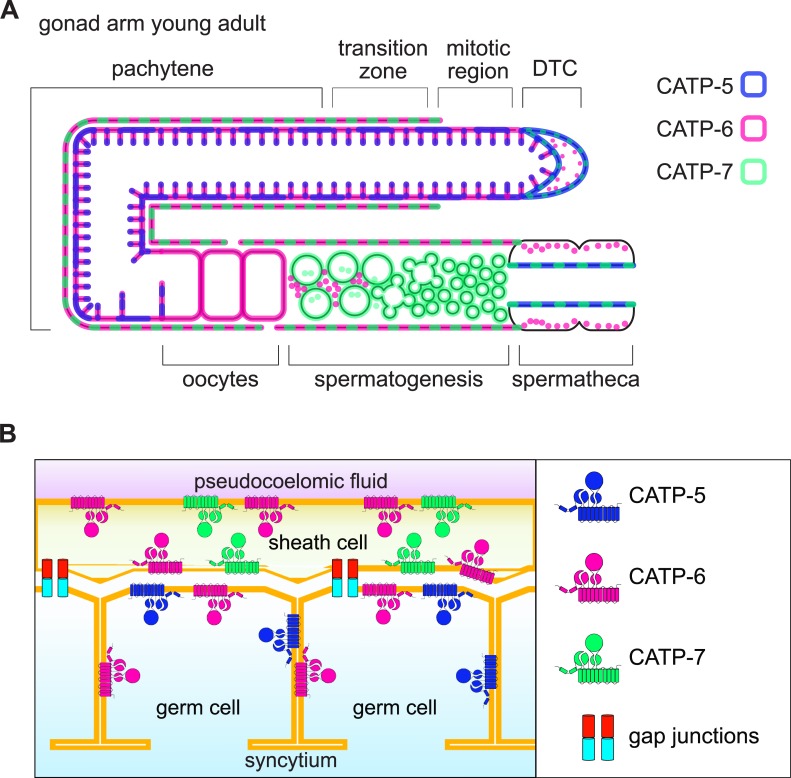
Schematic overview of P5B ATPase expression in somatic gonad and germ line. (A) CATP-5, CATP-6 and CATP-7 expression in somatic gonad and germ line in a young adult hermaphrodite. Most of the ovary is contiguously covered by somatic sheath cells; however, there this coverage ceases prior to the distal tip cell. In addition, the proximal sheath cells are perforated by "sheath pores" 100–200 nm in diameter, allowing contact between oocytes that have pinched off the syncytium and the pseudocoelomic fluid [46]. Dashed lines: 2 paralogs expressed in the same subcellular compartment. (B) Schematic of P5B localization in the distal oviduct (pachytene) region of the gonad.

All three paralogous P5B ATPases are strongly expressed in the germ line and the somatic gonadal tissues (Fig 10A). Since the gonadal sheath cells and distal tip cell are known to be physiologically coupled to the germ line ([45,46], Fig 10A and 10B), we considered it likely that the P5Bs would have essential functions during gonad development and germline proliferation. To test this hypothesis, we obtained and/or generated single, double and triple null mutant strains for *catp-5*, *catp-6* and *catp-7*.

The single null mutants are all homozygous viable and fertile. *catp-5(tm4481)* is a 705 bp deletion that removes the coding sequences for 235 aa of M3 and M4 including the putative substrate binding site, the phosphorylation site (P domain) within the large intracellular loop, plus half of the N domain and also results in a frameshift (Fig 1). *catp-5(0)* animals have an overtly wt phenotype, as expected [11], with brood size and growth rate similar to those of wt N2 (Table 1, Table 2).

*catp-6(ok3473)* is a deletion of 934 bp that removes the coding sequences for 281 aa of M5, M6 and M7, including half of the P domain and one Mg^2+^ coordination motive (GDGAND), followed by a frameshift (Fig 1). *catp-6(0)* mutants are Egl (egg-laying defective), Unc (uncoordinate; sluggish) and Gro (slow growth). *catp-6(0)* mutants present a highly significant smaller brood size when compared to N2 (Table 1, group c, ANOVA, Tukey post-hoc test, adj. *p*-value = 0.000000416). Only 63% of the progeny of *catp-6(0)* animals reached L4 stage after 48 h, which is highly significant different to wt N2 (Table 2, group y, Fisher's exact test, adj. *p*-value = 0.0000000000705).

*catp-7(tm4438)* is a deletion of 1222 bp (plus 6 bp insertion) that removes the coding sequence for 384 aa and therefore most of the large cytoplasmic loop between the N domain (ATP binding site) and the P domain (Mg^2+^ coordination motive TGDN), but does not result in a frame shift (Fig 1). *catp-7(0)* single mutants are overtly wt, with brood size and growth rate the same as wt N2 (Table 1, Table 2).

*catp-7(0)*; *catp-5(0)* double mutants appear very similar to wild type. Mid L4, young adults and 1 day old adults do not have any obvious phenotype in terms of somatic gonad development or germline proliferation.

**Fig 11:**
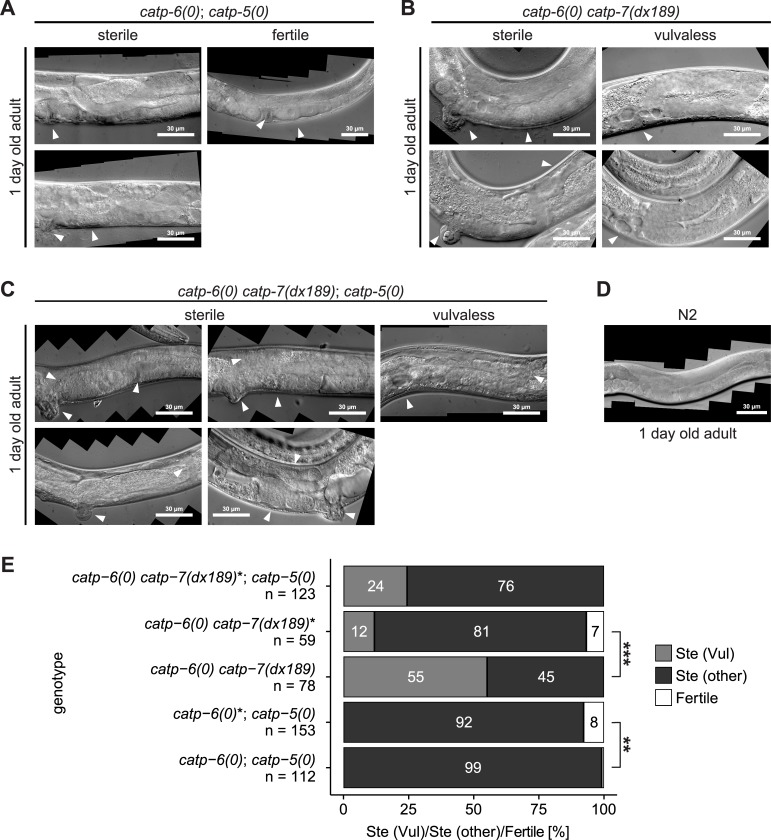
Phenotypic analysis of single, double and triple null mutants. (A-C) 1 day old adult gonad of: *catp-6(0)*; *catp-5(0)* double mutants (*catp-6(ok3473)* IV; *catp-5(tm4481)* X), *catp-6(0) catp-7(dx189)* double mutants (*catp-6(ok3473) catp-7(dx189[delta 1492 bp Ma-M3 + gfp + loxP]* IV) and *catp-6(0) catp-7(dx189)*; *catp-5(0)* triple mutants (*catp-6(ok3473) catp-7(dx189[delta 1492 bp Ma-M3 + gfp + loxP]* IV; *catp-5(tm4481)* X). (D) 1 day old adult gonad of wt N2. (E) Quantification of phenotypic segregants. (Ste (Vul)): Sterile Vulvaless; Ste (other): Sterile Evl, Pvl or Muv. Fertile: Able to produce progeny (normal vulva). Asterisk indicates that animals were obtained from a hermaphrodite heterozygous for the mutant chromosome. Fisher's exact test and subsequent Holm’s correction for multiple comparison, adj. *p*-value: ** ≤ 0.01, *** ≤ 0.001.

*catp-6(0); catp-5(0)* double mutants are nearly completely sterile (Ste). Therefore, these worms were obtained from a balanced strain heterozygous for *catp-6(0)* (*catp-6(ok3473)*/ *nT1[qIs51]* (IV;V); *catp-5(tm4481)*). Among the double mutant progeny obtained from this strain, we found that most (Fig 11E, 92%; n = 153) were sterile; these animals acquired a Pvl phenotype (Protruding vulva) as they aged (Fig 11A and 11E). The remaining fraction (8%) of the double mutants were fertile (Fig 11E) and appeared essentially wild type. However, nearly all of the progeny of these worms (99% (n = 112) were sterile (Fig 11E). Since the sterile phenotype is more severe when the progeny are derived from a hermaphrodite homozygous for *catp-6(0)*, this suggests that *catp-6(+)* probably provides maternal rescue (Fisher's exact test, adj. *p*-value = 0.00896; paternal rescue is also possible, but this is less likely). Young adult *catp-6(0); catp-5(0)* animals have a fully developed gonad, but the number of germ cells is smaller than in wild type (S1 Fig). These animals possessed both sperm and oocytes at this stage (S1 Fig). Mid to late L4-stage *catp-6(0); catp-5(0)* animals showed no obvious phenotype in terms of DTC migration (S1 Fig).

As one-day-old adults, *catp-6(0); catp-5(0)* animals are obviously different from wt N2. The DTCs inappropriately continue to migrate, leading to abnormal gonad arm positions (Fig 11A). Although pachytene exit was not blocked, the growth of late stage oocytes was impaired and, unlike wild type worms, the first ovulation had not yet taken place (Fig 11A). A small fraction of the *catp-6(0); catp-5(0)* animals are fertile (1%), but these have a greatly reduced brood size, ranging from 1 to 19 progeny (Fig 11A and 11E).

*catp-6* and *catp-7* are separated by only 0.52 cM on chromosome IV, and we were not successful in obtaining the *catp-6(0) catp-7(0)* double mutant by meiotic recombination. Therefore, we used CRISPR/Cas9 to generate a null allele of *catp-7* in the background of *catp-6(0)*. We exchanged 1492 bp between exon 2 and exon 5 of isoform *catp-7a* with the coding sequence of *gfp*. The deletion removes the coding sequences for Ma to M3, the A domain (dephosphorylation motif TGE) and results in a subsequent frameshift (*catp-7(dx189)*, Fig 1). Therefore, *catp-7(dx189)* is not able to encode a functional transporter protein. Since we found that *catp-6(0) catp-7(dx189)* animals are usually sterile (Ste); the double mutant chromosome was maintained over a balancer (*catp-6(ok3473) catp-7(dx189)*/ *nT1[qIs51]* (IV;V)).

Homozygous *catp-6(0) catp-7(dx189)* worms that are derived from a heterozygous hermaphrodite display a high fraction of sterility (81% n = 59). These animals also typically exhibit abnormal vulva phenotypes, including Pvl, Evl (everted vulva) and Muv (Multivulva). Furthermore, 12% (n = 59) of *catp-6(0) catp-7(dx189)* were Vul (vulvaless). Homozygous *catp-6(0) catp-7(dx189)* worms derived from a homozygous hermaphrodite, are more severely affected: 55% (n = 78) are Vul (Ste (Vul)) and 100% (n = 78) are sterile (Ste (other), Fig 11E). This suggests that *catp-6(+)* and/or *catp-7(+)* probably confer maternal rescue of these gonadal defects (Fisher's exact test, adj. *p*-value = 0.000000108).

Mid to late L4 *catp-6(0) catp-7(dx189)* worms exhibit a delay in DTC migration when compared to wt L4 of the same stage (S2 Fig), probably due to reduced germline proliferation. Young adult *catp-6(0) catp-7(dx189)* animals often show a fully reflexed gonad (S2 Fig). However, in contrast to wild type, no gametogenesis has yet taken place in the proximal arm (S2 Fig). Roughly half of analyzed gonads exhibit a DTC migration defect by omitting the second turn towards the midsection (S2 Fig and Fig 11B). 1 day old adult *catp-6(0) catp-7(dx189)* worms have often completed spermatogenesis, but oogenesis and ovulation are typically delayed (Fig 11B). The overall number of germ cells is usually much smaller than in wild type, suggesting a defect in germline proliferation.

We used DAPI staining of extruded gonads to determine whether gonadal sheath cells were still present in animals with defective germline proliferation. We were not able to unambiguously determine the exact number of sheath cell nuclei per gonad arm; however, we consistently observed multiple sheath cell nuclei (S3 Fig), thus indicating that the mutant phenotype does not result from a complete absence of gonadal sheath cells.

We obtained animals of genotype *catp-6(0) catp-7(dx189); catp-5(0)* from hermaphrodites of genotype *catp-6(ok3473) catp-7(dx189)*/ *nT1[qIs51]* (IV;V); *catp-5(tm4481)*. All of the *catp-6(0) catp-7(dx189); catp-5(0)* animals produced by this strain were sterile. Furthermore, 76% (n = 123) had an abnormal vulva phenotype—everted, protruding, or multivulva (Ste (other)) and 24% (n = 123) were Vul (Ste (Vul), Fig 11E and 11C).

Mid to late L4 *catp-6(0) catp-7(dx189); catp-5(0)* animals showed a strong delay in DTC migration when compared to wt from the same stage (S4 Fig). The number of germ cells is severely diminished, suggestive of a proliferation defect (S4 Fig).

Among 1 day old adult triple mutants, some were able to produce both sperm and oocytes, but the oocytes were relatively small and abnormal in appearance. These animals also occasionally exhibited abnormal distal germline proliferation (Fig 11C), and some also had proximal germline proliferation. Most of the animals displayed severe defects in DTC migration with multiple inappropriate turns and perpetual DTC migration. Vul animals often had a large cavity in the midsection, consistent with the formation of at least a partial uterus in the absence of a vulva (Fig 11C).

## Summary and discussion

### Spatial and temporal expression patterns

We find that the *C*. *elegans* P5B ATPases are expressed in a wide range of tissues. Within these tissues, they exhibit both dynamic and overlapping expression patterns. These are summarized in Table 3 and Fig 10A. Depending upon context, each of the P5Bs may localize either to internal membranous structures or to the plasma membrane. However, each protein has distinct targeting signals, e.g., CATP-5 and CATP-7 both localize to the apical plasma membrane in spermathecal epithelial cells, but in the excretory cell CATP-7 is on the basolateral plasma membrane, whereas CATP-5 localizes to vesicles and the apical/lumenal membrane.

**Table 3:** Summary of expression and localization of nematode P5Bs.

	CATP-5	CATP-6	CATP-7
**Intestine (L1)**	Apical brush border plasma membrane	Apical brush border plasma membrane	Apical brush border plasma membrane
**Intestine (adults)**	Apical brush border plasma membrane	Basolateral and subapical tubular/vesicular	Basolateral and subapical tubular/vesicular
**Pharyngeal muscle**	Not detectable	Cortical vesicles	Plasma membrane. T-tubules(?)
**Neurons**	Apical plasma membrane (amphids)	Cortical vesicles	Apical plasma membrane (amphids)
**Germ line (adults)**	Plasma membrane in hermaphrodite distal and pachytene regions	Plasma membrane in hermaphrodite distal and pachytene regions	Not detectable
**Germ line (spermatogenesis)**	Not detectable	Vesicular in spermatocytes. Cytoplasmic in spermatids.	Plasma membrane in spermatocytes. FB-MOs in spermatids.
**DTC**	Plasma membrane	Cortical vesicles	Plasma membrane and vesicles
**Developing vulva (L4)**	Not detectable	Cortical vesicles	Plasma membrane
**Central somatic** **gonad (early L4)**	Not detectable	Cortical vesicles	Plasma membrane
**Spermatheca**	Apical plasma membrane	Basolateral cortical vesicles.	Apical plasma membrane
**Z1 and Z4**	Not detectable	Cortical vesicles	Plasma membrane and vesicles
**Excretory cell** **(larvae and adults)**	Vesicular, luminal membrane	Not detectable	Basolateral plasma membrane
**Hypodermis** **(adults)**	Not detectable	Not detectable	Plasma membrane
**Gonadal sheath** **(adults)**	Not detectable	Plasma membrane rafts and between rafts	Plasma membrane rafts

### Overlapping functions in reproductive tissues

Each of the single mutants, *catp-5(0)*, *catp-6(0)* and *catp-7(0)* is viable and fertile. However, we observed distinct synthetic sterile phenotypes in the *catp-6(0); catp-5(0)* and *catp-6(0) catp-7(dx189)* double mutants. We do not observe any mutant phenotypes in the CRISPR/Cas9 tagged strains (*catp-6*::*mKate2* IV, *gfp*::*catp-5* X, or *catp-6*::*mKate2 gfp*::*catp-7* IV double mutants (Table 1, Table 2)), therefore we conclude that the *fp*-tagged alleles are functional.

*catp-6(0); catp-5(0)* double mutants, have nearly normal somatic gonad development, but exhibit delayed germline proliferation and have very small brood sizes. Since both CATP-5 and CATP-6 are present on the plasma membrane of pachytene-stage germ cells, the simplest interpretation is that these two proteins function redundantly within this tissue to take up a common substrate from the extracellular fluid. However, CATP-6 is also expressed on the plasma membrane of the gonadal sheath cells, which are physiologically connected to the pachytene-stage germ line via numerous gap junctions [45]. Thus, the germ line should be able to obtain P5B transport substrates either by direct uptake or by transfer from the sheath cells (Fig 10B). Additional work will be necessary to resolve the basis for the sterility of the *catp-6(0); catp-5(0)* double mutant.

*catp-6(0) catp-7(dx189)* double mutants exhibit variably severe defects in somatic gonad development, with uterine, spermathecal and sheath cells under-represented or absent. Given the known importance of the sheath cells for germline proliferation, their absence would be sufficient to explain the sterile phenotype of *catp-6(0) catp-7(dx189)* double mutants. However, we observed impaired germline proliferation even in animals where multiple sheath cells were present, so it is possible that sheath cell function is defective in *catp-6(0) catp-7(dx189)* animals.

The basis for the vulvaless phenotype of *catp-6(0) catp-7(dx189)* animals is also currently unresolved. The anchor cell could be missing and/or it could be defective in its ability to produce the inductive LIN-3 signal [47,48]. Furthermore, since CATP-6::mKate2 and GFP::CATP-7 are both expressed in the developing vulval epithelial cells, it could be that the VPCs of double mutants are not competent to respond to LIN-3.

### Distal tip cell migration phenotype

Although distal tip cell (DTC) migration is normal in each of the P5B single mutants, double and triple mutants show characteristic defects in migration of the DTCs. The perpetual DTC migration defect observed in *catp-6(0)*; *catp-5(0)* double mutants is very similar to that observed in *vab-3(lf)* mutants, in which the alpha integrin *ina-1* is not downregulated [49,50]. Furthermore, in *catp-6(0) catp-7(dx189)* double mutants, the DTCs frequently omit the second turn towards the center of the animal; this phenotype is very similar to that observed in mutants where either one of the integrin-associated proteins, SRC-1 or talin, is defective [51,52].

We speculate that *catp-5*, *catp-6* and *catp-7* function redundantly within the DTCs to allow correct localization and/or function of integrins and integrin-associated proteins. Given the known association between P5B ATPase function and Parkinsons disease, it may be significant that the human ortholog of *vab-3* is Pax6, which has been shown to be important for dopaminergic neuron survival [53]. Moreover, the expression of integrin α5β1 is crucial for dopaminergic neurite outgrowth to the striatal cell region [54]. Since α5β1 integrin is known to be a target of Pax6 [55], we speculate that this is a conserved regulatory interaction, and that P5B ATPases may act coordinately to regulate integrin function.

### P5B subcellular localization

Mammalian P5B ATPases have been reported to localize to lysosomes, late endosomes, multivesicular bodies, (ATP13A2; [9,56–58] and ER (ATP13A4, [59]). While there are hints that ATP13A3 may localize to the plasma membrane [60], this has not been definitively established. We find that each of the nematode P5Bs can localize to the plasma membrane in certain tissues and stages of development. We observed that the CATP-6 localization pattern in spermathecal epithelial cells exhibits obvious similarity to that of the early endosomal marker RFP::RAB-5. Higher resolution imaging will be necessary to confirm that RAB-5 and CATP-6 colocalize at the level of individual vesicles. We did not observe substantial localization of CATP-6 to vesicular compartments marked by LMP-1 (lysosomes), RME-1 (recycling endosomes), RAB-11 (recycling endosomes), HGRS-1 (early endosomes), SS::GFP::CD4-HCIMPR (endosomes) or TGN-38 (trans-Golgi network). Therefore, we do not find strong evidence that the nematode P5Bs function primarily within lysosomes.

### P5B substrate specificity

Although the transport specificity of P5B ATPases has not been unambiguously determined, various candidates have been proposed based on biological evidence. In the yeast, *Saccharomyces cerevisaie*, loss of P5B (Ypk9) function results in hypersensitivity to Mn^2+^, leading to the suggestion that Ypk9 detoxifies Mn^2+^ by pumping it into the vacuole. In the slime mold, *Dictyostelium discoideum*, loss of P5B (Kil2) function prevents Mg^2+^-dependent killing of phagocytosed bacteria, leading to the suggestion that Kil2 pumps Mg^2+^ into the phagosome. In mammalian tissue culture cells, loss of ATP13A2 function results in overaccumulation of Zn^2+^, suggesting that this P5B might play a role in Zn^2+^ homeostasis [57]. On the other hand, ATP13A3 has recently been implicated as a candidate polyamine transporter [60]. With regard to the nematode P5Bs, the data of Heinick et al. [11] strongly suggest that polyamines are transported from the gut lumen into the intestinal cells via CATP-5 expressed on the plasma membrane. If CATP-6 and CATP-7 have the same specificity as CATP-5, then this would provide a simple explanation for their functional overlap within the germ line and the somatic sheath cells. This would also suggest that the sheath cells and the germ line are unable to autonomously produce sufficient quantities of polyamines to enable normal development and function of the reproductive system. Furthermore, given our original identification of CATP-6 as an interactor of the GON-2 Mg^2+^ channel, it is possible that the P5Bs function as antiporters, exchanging polyamines and Mg^2+^ between compartments.

## Supporting information

S1 Fig L4s and young adult *catp-6(0)*; *catp-5(0)* double mutants. *catp-6(ok3473)* IV; *catp-5(tm4481)* X.
(EPS)

S2 Fig L4s and young adult *catp-6(0) catp-7(dx189)* double mutants. *catp-6(ok3473) catp-7(dx189[delta 1492 bp Ma-M3 + gfp + loxP]* IV.
(EPS)

S3 Fig DAPI staining of sheath cell nuclei of *catp-6(0) catp-7(dx189)* double mutants. *catp-6(ok3473) catp-7(dx189[delta 1492 bp Ma-M3 + gfp + loxP]* IV.
(EPS)

S4 Fig L4s and young adult *catp-6(0) catp-7(dx189)*; *catp-5(0)* triple mutants. *catp-6(ok3473) catp-7(dx189[delta 1492 bp Ma-M3 + gfp + loxP]* IV; *catp-5(tm4481)* X.
(EPS)
